# Endpoint Recombinase Polymerase Amplification (RPA) Assay for Enumeration of Thiocyanate-degrading Bacteria

**DOI:** 10.1264/jsme2.ME21073

**Published:** 2022-03-08

**Authors:** Mamoru Oshiki, Toshikazu Fukushima, Shuichi Kawano, Junichi Nakagawa

**Affiliations:** 1 Department of Civil Engineering, National Institute of Technology, Nagaoka College, Japan; 2 Division of Environmental Engineering, Faculty of Engineering, Hokkaido University, North 13, West 8, Kita-ku, Sapporo, Hokkaido 060–8628, Japan; 3 Advanced Technology Research Laboratories, Research & Development, Nippon Steel Corporation, Japan; 4 Department of Computer and Network Engineering Graduate School of Informatics and Engineering, The University of Electro-Communications, Japan

**Keywords:** recombination amplification reaction (RPA), endpoint assay, gene quantification, thiocyanate dehydrogenase

## Abstract

An endpoint recombination amplification reaction (RPA) assay for assessing the abundance of the gene encoding thiocyanate dehydrogenase (TcDH) in *Thiohalobacter* has been developed. The RPA reaction was performed at 37°C for 30‍ ‍min, terminated by the addition of sodium dodecyl sulfate (SDS) solution, and the DNA concentration of the RPA product was fluorometrically measured. The abundance of TcDH in 22 activated sludge samples and 7 thiocyanate-degrading enrichment cultures ranged between 2.5×10^3^ and 1.5×10^6^ copies μL^–1^, showing a linear relationship (*R^2^*=0.83) with those measured using a conventional quantitative PCR assay.

Biological wastewater treatments, such as the activated sludge process, are widely used, and measurements of the abundance of functional (*e.g.*, nitrifiers and denitrifiers) and pathogenic microorganisms are important for assessing the performance and efficiency of these processes (Ishii and Sadowsky, 2008; Nurmiyanto *et al.*, 2017; Okamura *et al.*, 2018). Various molecular tools have been developed for this purpose, including fluorescence *in-situ* hybridization (FISH) and polymerase chain reaction (PCR)-based assays. Quantitative PCR (qPCR) assays (*i.e.*, real-time PCR assays) have been widely used to evaluate the abundance of taxonomic and functional genes due to their high sensitivity and reliability (Harms *et al.*, 2003; da Silva *et al.*, 2007). The amplification of DNA molecules during qPCR assays is monitored by increases in fluorescence derived from a DNA-intercalation dye (*e.g.*, SYBR Green I) or specific fluorescence probe using a real-time thermal cycler (Smith and Osborn, 2009). However, conventional qPCR assays have the following limitations: 1) these assays require a real-time thermal cycler, which is expensive (generally >20,000 USD) and generally not portable, and 2) the PCR reaction requires a thermal cycler to control reaction temperatures for the denaturing of DNA molecules (generally at 95°C), annealing of oligonucleotide primers (55–60°C), and extension (68–75°C). Besides qPCR assays, endpoint PCR assays, including a competitive PCR assays, may be employed to measure the abundance of a target DNA in a sample (Schmittgen *et al.*, 2000; Dionisi *et al.*, 2002; Rudney *et al.*, 2003). An advantage of the endpoint PCR assay is its simplicity (*i.e.*, it does not require a real-time thermal cycler); however, a thermal cycler is still needed for temperature control.

Recombinase polymerase amplification (RPA) (Piepenburg *et al.*, 2006) is a relatively novel molecular technique that amplifies specific DNA molecules as well as PCR amplification. In the RPA reaction, DNA molecules are amplified under isothermal conditions using 2 oligonucleotide primers and the following 3 enzymes: endonuclease IV, exonuclease II, and strand displacing DNA polymerase (Lobato and O’Sullivan, 2018). A marked difference between the RPA and PCR reactions is that the RPA reaction is an isothermal reaction that occurs at 37–42°C; therefore, a thermal cycler is not required. A real-time RPA reaction, in which the amplification of DNA molecules is monitored by increases in the fluorescence intensity of DNA-intercalation dye using a real-time thermal cycler, was previously developed as a detection system of environmental microorganisms (mainly pathogens) (Zhu *et al.*, 2018). However, the real-time RPA assay still requires a real-time thermal cycler, which limits its application to on-site measurements.

Therefore, the present study aimed to develop an “endpoint” RPA assay as a reliable method for measuring the abundance of functional genes in the environment. As described above, the RPA reaction is an isothermal reaction provided using a heat block or water bath, and portable fluorometers (*e.g.*, Qubit fluorometer) (Thermo Fisher Scientific) are commercially available. The abundance of functional genes may be measured using the endpoint RPA assay, even on-site; however, limited information is currently available on the sensitivity and reliability of this assay. Therefore, we developed an endpoint RPA assay to measure the gene encoding thiocyanate dehydrogenase (TcDH) in activated sludge and bacterial enrichment cultures. Thiocyanate (SCN^–^) is a common contaminant in coke-oven wastewater and mining wastewater (Toh and Ashbolt, 2002), and TcDH is involved in the degradation of SCN^–^ into cyanate and ammonia (Tikhonova *et al.*, 2020). We recently operated SCN^–^-degrading bioreactors and enriched the SCN^–^-degrading bacterium, *Thiohalobacter* sp. strain FOKN1 (Oshiki *et al.*, 2019). A reliable method to monitor the abundance of FOKN1 in activated sludge is valuable for examining the performance of SCN^–^ removal in the activated sludge process. Therefore, we aimed to develop an endpoint RPA assay to assess the abundance of the *Thiohalobacter* TcDH gene in activated sludge and SCN^–^-degrading enrichment cultures. In the endpoint RPA assay, the termination of the RPA reaction at the end of the reaction is essential for quantitative applications, and the influence of potential inhibitors of the RPA reaction was investigated. Additionally, the influence of the incubation time on sensitivity was examined.

To evaluate the *Thiohalobacter* TcDH gene using the endpoint RPA assay, total genomic DNA was extracted from 22 activated sludge samples and 7 SCN^–^-degrading enrichment cultures. Activated sludge samples were collected from a 3.4-L moving bed biofilm reactor fed with synthetic coke-oven wastewater. The reactor was operated at pH 7.5 and 30°C, and coke-oven wastewater containing 4‍ ‍mM of SCN^–^, 31‍ ‍mM NH_4_^+^, 1.4‍ ‍mM phenol, and 2.3‍ ‍mM thiosulfate was continuously supplied into the reactor at a loading rate of 3.5 to 10.5 L day^–1^. Activated sludge samples were collected twice a week, and sludge pellets were stored at –80°C for DNA extraction. Details on cultivation conditions and medium compositions in the enrichment culture are available in our previous study (Oshiki *et al.*, 2019). SCN^–^-degrading bacteria were enriched from activated sludge using inorganic medium containing 3.44‍ ‍mM SCN^–^. The culture was aerobically incubated at 30°C, and 2% of the culture was transferred into fresh medium when SCN^–^ was completely consumed in the culture. The subculture was repeated 8 times in total, and a portion of the culture was collected at each subculture (except for the 1^st^ subculture) for DNA extraction. Genomic DNA was extracted using the DNeasy PowerSoil kit (Qiagen) according to the instruction manual supplied by the company, and was then subjected to the endpoint RPA assay.

The endpoint RPA assay was performed in 10‍ ‍μL of the RPA reaction mixture containing 1‍ ‍ng of an extracted DNA sample or 1‍ ‍μL of a standard DNA sample (10^7^ to 10^1^ copies μL^–1^), forward and reverse oligonucleotide primers (0.96‍ ‍μM each), 0.5‍ ‍μL of 0.28 M magnesium acetate, and 1× TwistAmp Basic reagent (TwistDx). The oligonucleotide primers used, TcDH_F1 (5′–AGTATGTCGCCTTCGCCGACGGCCAGAAGGAC–3′) and _R1 (5′–CCAGCGGCAGCTCGGGATGCCAGGTGAAGGCGTC–3′), were designed in the present study and amplified a partial sequence of the *Thiohalobacter* TcDH gene (281 bp). The mixture was prepared and incubated in triplicate at 37°C for 10 to 30‍ ‍min using the thermal cycler PC802 (ASTEC). The standard curve needed to quantify the copy numbers of the *Thiohalobacter* TcDH gene in the tested samples was prepared using a dilution series of standard DNA samples. Standard DNA was prepared by amplifying the 3.2-kb region (nucleotide position on the genome sequence; 559,381–562,591) of the *Thiohalobacter* sp. FOKN1 genome (accession number; AP018052), including the region encoding TcDH (nucleotide position; 559,963–561,429), by conventional PCR. The PCR mixture had a volume of 25‍ ‍μL and contained 2.5‍ ‍ng of an extracted DNA sample, oligonucleotide primers (0.2‍ ‍μM each), dNTPs (200‍ ‍μM), 1× PCR buffer, and PrimeStar GXL polymerase (0.025‍ ‍U μL^–1^) (TakaraBio). The oligonucleotide primers used were FOKN1_F (5′–ATGCTCGCACTCGCTGCTCCGGGCACTGCAGTGGCCGATAGCATCGACGGAAACTTCCCC–3′) and _R (5′–CTACCAGTTACACGGGCCCTGGCTGCGGTCGGGGCACACGGTCAGCGAATCCGTCACCGC–3′), which were designed from the FOKN1 genome sequence and had no primer-template mismatches. Cycling conditions were as follows: 35 cycles at 98°C for 10‍ ‍s, 55°C for 15‍ ‍s, and 68°C for 3‍ ‍min, and finally 68°C for 10‍ ‍min. The PCR product was purified using the FastGene Gel/PCR Extraction Kit (Nippon Genetics), and DNA concentrations were measured fluorometrically as described below. The purified PCR product was serially diluted with distilled water in the range of 10^7^ to 10^1^ copies μL^–1^.

Prior to the endpoint RPA assay on TcDH, a treatment that terminates the RPA reaction after a specific incubation period needs to be developed because the RPA reaction is an isothermal reaction that occurs at 37 to 42°C and will continue even after the end of the incubation (*i.e.*, room temperature). Therefore, we added SYBR Green I and one of the following potential inhibitors to the RPA mixture containing the standard DNA sample (2.6×10^5^ copies μL^–1^): 80% (v/v) H_2_SO_4_, 10 N NaOH, 10% (w/v) sodium dodecyl sulfate (SDS), and saturated phenol solution, and the occurrence of DNA amplification by the RPA reaction was examined. The occurrence of DNA amplification was judged by monitoring increases in the fluorescence intensity of SYBR green I using the real-time thermal cycler MiniOpticon (Bio-Red). When the above potential inhibitors were not added, increases in fluorescence intensity occurred after 6.1±0.45‍ ‍min of the incubation. These increases continued until approximately 12‍ ‍min and then plateaued, similar to the conventional quantitative PCR (qPCR) assay. The specific amplification of the *Thiohalobacter* TcDH gene was ascertained by agarose gel electrophoresis (1.5% agarose) using a 100-bp DNA ladder marker (Nippon Genetics), and no specific band appeared from the sample without the addition of any DNA template. When we added 0.5‍ ‍μL of the H_2_SO_4_, NaOH, or SDS solution separately to 10‍ ‍μL of the RPA mixture, no increase in fluorescence intensity occurred even after 30‍ ‍min of the incubation, indicating that the addition of the above chemicals inhibited the RPA reaction. On the other hand, the addition of phenol did not inhibit the RPA reaction, and the increase in fluorescence intensity was detectable after 6.7±0.47‍ ‍min of the incubation. These results indicated that the RPA reaction was inhibited by H_2_SO_4_, NaOH, and SDS. Since H_2_SO_4_ and NaOH are highly hazardous chemicals, the addition of SDS solution was selected as a method to terminate the RPA reaction. In our endpoint RPA assay, 0.5‍ ‍μL of 10% (w/v) SDS solution was added to the RPA reaction mixture at the end of the incubation to terminate the RPA reaction, and 1‍ ‍μL of the RPA product was subjected to the fluorometric assessment of DNA concentrations using the Qubit dsDNA BR assay kit and Qubit 3 fluorometer (Thermo Fisher Scientific). The RPA product was mixed in a 0.5-mL thin-walled plastic tube (Thermo Fisher Scientific) with 199‍ ‍μL of 1× Qubit working solution including the Qubit dsDNA BR reagent. After mixing by gentle inversion, the tube was incubated at room temperature for 5‍ ‍min. The fluorescence intensity of the tube was measured using the Qubit 3 fluorometer according to the instruction manual provided by the supplier. The fluorometer provided the intensity of green fluorescence as relative fluorescent units (RFU).

Sensitivity and reliability were examined by performing the endpoint RPA assay using a dilution series of standard DNA (the initial copy number of *Thiohalobacter* TcDH ranged between 2.6×10^1^ and 10^7^ copies μL^–1^ in the reaction mixture), and the reaction was terminated after 10, 20, and 30‍ ‍min of the incubation. As shown in Fig. 1, the highest sensitivity was achieved when the RPA reaction was performed for 30‍ ‍min, and increases in fluorescence intensity were detectable in samples with an initial concentration of 2.6×10^2^ copies μL^–1^ in the reaction mixture. A linear relationship (*R^2^*=0.98) was observed between the initial copy number and measured fluorescence intensity in the range of 2.6×10^2^ to 10^5^ copies μL^–1^. Therefore, the incubation time was set to 30‍ ‍min in the endpoint RPA assay described below.

The abundance of the *Thiohalobacter* TcDH gene in activated sludge (*n*=22) and SCN^–^-degrading enrichment cultures (*n*=7) was assessed by the developed endpoint RPA assay using the standard curve prepared above and by performing conventional qPCR assays. The PCR mixture had a volume of 20‍ ‍μL and contained 2‍ ‍ng of an extracted DNA sample or 2‍ ‍μL of standard DNA (10^7^ to 10^1^ copies μL^–1^), the oligonucleotide primers TcDH_F1 and TcDH_R2 (0.4‍ ‍μM each), and 1×KAPA SYBR fast universal reagent (Nippon Genetics). Cycling conditions were as follows: 95°C for 3‍ ‍min; 40 cycles at 95°C for 3‍ ‍s, and 60°C for 30 s; and finally, 65 to 95°C with increments of 0.5°C for a melting curve ana­lysis. The qPCR assay was conducted in triplicate on the real-time thermal cycler. As shown in Fig. 2, the copy numbers of TcDH assessed by the endpoint RPA assay were similar to those by the qPCR assay. The linear correlation (*R^2^*=0.83) observed between the copy numbers assessed by the endpoint RPA and qPCR assays indicated that the developed endpoint RPA assay enabled us to approximate the copy numbers of TcDH.

We herein demonstrated that the endpoint RPA assay allowed us to approximate the copy number of TcDH in activated sludge and SCN^–^-enrichment cultures. The endpoint RPA assay has mostly been used to detect the presence or absence of specific microorganisms (mostly pathogens) (Kersting *et al.*, 2014; Crannell *et al.*, 2016; Hieno *et al.*, 2021), whereas its application as a quantitative assay remains limited. In the quantitative endpoint RPA assay, the termination of the RPA reaction after a certain incubation period is essential; however, there is currently no method available for this purpose. The present results showed that the addition of SDS solution halted the RPA reaction, and expanded the potential applications of the endpoint RPA assay to measurements of the abundance of specific microorganisms. A quantitative endpoint RPA assay for on-site measurements requires isothermal conditions and a portable fluorometer. Regarding isothermal conditions, the simplest approach to maintain the incubation temperature required for the RPA reaction in a field survey is to use a commercial Styrofoam box with warmed water. The RPA reaction using the TwistAmp Basic reagent occurs at 37–42°C, and the incubation temperature needs to be maintained in this range. Portable fluorometers, such as the Qubit 3 fluorometer, are commercially available, and fluorometric measurements using the Qubit 3 fluorometer (electricity consumption; 100‍ ‍Wh) may be performed using a portable lithium-ion power battery, which enables the abundance of specific microorganisms to be measured, even outdoors. Our endpoint RPA assay requires a 30-min incubation and another 10‍ ‍min to terminate the RPA reaction and measure fluorescence intensity. The running cost of the endpoint RPA assay is 2.7 USD per assay, including reagent costs for the RPA reaction and fluorometric measurements of DNA concentrations. This analytical time and running cost are similar to those of qPCR assays. The present results indicate the applicability of the endpoint RPA assay as a reliable method for measurements of the abundance of TcDH in the environment.

In addition to RPA, loop-mediated isothermal amplification (LAMP) amplifies specific DNA molecules under isothermal conditions, and has been applied to detect and quantify the abundance of taxonomic and functional genes in the environment (Aoi *et al.*, 2006; Nathaniel *et al.*, 2019). An advantage of the RPA reaction is that the design of the oligonucleotide primer set used for the RPA reaction is markedly easier than that of LAMP. The RPA reaction requires 2 oligonucleotide primers as well as conventional PCR amplification, whereas LAMP requires 4–6 oligonucleo­tide primers (Nagamine *et al.*, 2002). Oligonucleotide primer coverage is critical for detecting the functional groups of microorganisms, while 6 conserved regions yielding appropriate amplicon sizes (<250 bp for LAMP amplification) are often missing. Therefore, the introduction of the endpoint RPA assay is easier than the LAMP assay. However, when designing oligonucleotide primers for the RPA reaction, the length of the primer used needs to be longer than that for conventional PCR amplification; typically 32–35 and 18–25‍ ‍bp, respectively (Li *et al.*, 2019). Although this requirement allows us to design an oligonucleotide primer set with higher specificity, it may be a bottleneck when we need to design a universal broad-coverage primer, such as the 515F primer, targeting most of the prokaryotic 16S rRNA gene sequence. Due to this technical limitation, the application of our proposed endpoint RPA assay may be preferable for assessing the abundance of specific functional genes involved in wastewater treatment and detecting specific pathogens. The application of the endpoint RPA assay targeting a functional gene other than TcDH needs to be examined using various types of samples, including soil and water samples.

## Citation

Oshiki, M., Fukushima, T., Kawano, S., and Nakagawa, J. (2022) Endpoint Recombinase Polymerase Amplification (RPA) Assay for Enumeration of Thiocyanate-degrading Bacteria. *Microbes Environ ***37**: ME21073.

https://doi.org/10.1264/jsme2.ME21073

## Figures and Tables

**Fig. 1.**
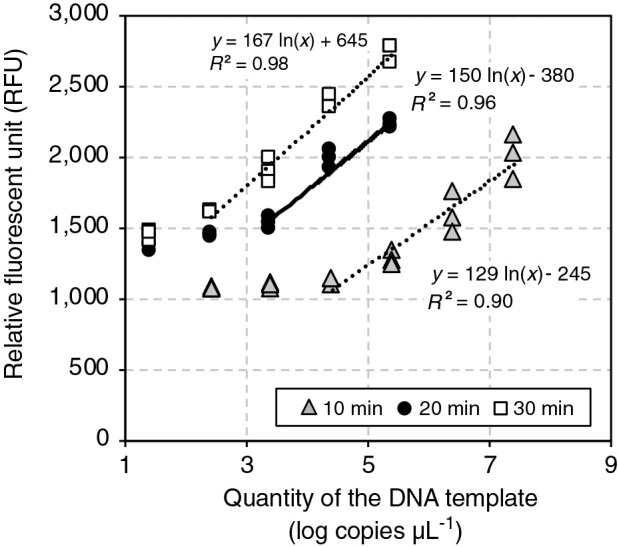
Influence of the incubation period on the quantitative detection of the thiocyanate dehydrogenase (TcDH) gene by the endpoint recombinase polymerase amplification (RPA) assay. The initial copy number of TcDH was in the range of 2.6×10^1^ to 10^7^ copies μL^–1^, and the RPA reaction was performed at 37°C. After an incubation for 10, 20, and 30‍ ‍min, the RPA product was subjected to the fluorometric assessment of DNA concentrations. DNA concentrations are shown as relative fluorescence units (RFU).

**Fig. 2.**
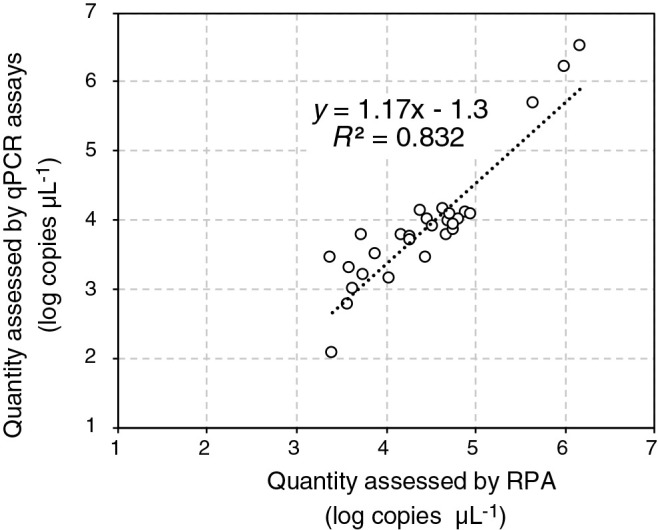
Correlation between the copy number of the thiocyanate dehydrogenase (TcDH) gene in activated sludge and enrichment cultures assessed by endpoint recombinase polymerase amplification (RPA) and quantitative PCR (qPCR) assays. Genomic DNA from 22 activated sludge samples and 7 SCN^–^-degrading enrichment cultures was subjected to assays, and a linear correlation (*R^2^*=0.832) was observed between the copy numbers of TcDH assessed by endpoint RPA and qPCR assays.
